# Memory retention following acoustic stimulation in slow-wave sleep: a meta-analytic review of replicability and measurement quality

**DOI:** 10.3389/frsle.2023.1082253

**Published:** 2023-05-11

**Authors:** Tylor J. Harlow, Matthew B. Jané, Heather L. Read, James J. Chrobak

**Affiliations:** ^1^Department of Psychological Sciences, University of Connecticut, Storrs, CT, United States; ^2^Department of Biomedical Engineering, University of Connecticut, Storrs, CT, United States

**Keywords:** meta-analysis, slow-wave sleep, slow oscillations, acoustic stimulation, memory, reliability, decline effect

## Abstract

The role of slow oscillations and spindles during sleep on memory retention has become an area of great interest in the recent decade. Accordingly, there are multiple studies that examine the efficacy of acoustic stimulation during sleep to facilitate slow oscillations and associated memory retention. Here, we run meta-analyses on a current set of 14 studies that use audible noise-burst sound stimulation to modulate overnight retention of word pairs (*k*_*S*_ = 12 studies, *k*_*ES*_ = 14 effect sizes, *n* = 206 subjects). Our meta-analyses demonstrate a steady, yearly decline in effect size that accounts for 91.8% of the heterogeneity between studies. We find that the predicted effect on memory retention in 2013 favored the acoustic stimulation condition at *d*_δ_ = 0.99 (95% CI [0.49, 1.49]), while the predicted effect in 2021 declined to a moderate and significant effect favoring no acoustic stimulation at *d*_δ_ = −0.39 (95% CI [−0.73, −0.05]). Our meta-regression model finds no coded study-level characteristics could account for the decline in effect sizes over time other than the publication date alone. Using available data, we estimate that 34% of subjects are not actually blind to the acoustic stimulation condition due to hearing acoustic stimulation during sleep. In addition, we find that the test-retest reliability of memory retention scores is nearly zero (*ρ*_*d*_ = 0.01, 95% CI [−0.18, 0.21]), and through simulation demonstrate the impact this has on statistical power and observed effect sizes. Based on our analyses, we discuss the need for larger sample sizes, true placebo controls, age range restrictions, open-data sharing, and improvements in the reliability of memory retention tasks.

## 1. Introduction

Converging lines of research support the theory that synchronous slow waves, spindles, and ripple oscillations and their coupling reflect the memory consolidation process that happens during sleep. In general, performance on declarative (verbal, event, and place) and non-declarative (sensory and motor skill) memory tasks improves following sleep (Barrett and Ekstrand, 1972; Plihal and Born, 1997; Mednick et al., 2003, 2011; Ellenbogen et al., 2007; Nishida and Walker, 2007; Miyamoto et al., 2017). An increase in the density of synchronous slow cortical oscillations (0.5–1.0 Hz) and thalamic spindles (12–15 Hz) during sleep is correlated with performance on various memory tasks following sleep (De Gennaro and Ferrara, 2003; Fogel and Smith, 2011; Niknazar et al., 2015; Cowan et al., 2020). The density of slow waves is highest during stages 2 and 3 of non-rapid eye movement (NREM) sleep (Stokes et al., 2022). In contrast, the density of fast spindle frequency oscillations is highest during stage 2 sleep when spindles are phase coupled with slow waves creating temporally coherent k-complex events (De Gennaro and Ferrara, 2003; Fogel and Smith, 2011; Stokes et al., 2022). Intracranially, slow waves arise from alternating phases of high and low spike-rate output from cortical neurons (Massimini et al., 2004; Steriade, 2006; Sanchez-Vives, 2020). In contrast, spindles are driven by thalamocortical circuits (Steriade, 2006). Thalamocortical spindles are phase coupled to the positive phase of cortical slow waves (Steriade, 2006; Diekelmann and Born, 2010). This coupling between slow waves and spindles positively correlates with improved sleep-dependent memory over the course of development and in adulthood (Hahn et al., 2020; Kurz et al., 2020). Computational models support a physiological mechanism where cortical output during the negative phase of the slow wave suppresses thalamic spindles and release from suppression in transition to the positive phase of slow waves promotes synchronous spindles (Mak-McCully et al., 2014). At the synaptic level, slow waves and spindles are thought to have complementary effects on synaptic plasticity which ultimately improves the signal-to-noise ratio of synapses and networks mediating memory (Tononi and Cirelli, 2006; Miyamoto et al., 2017). Spindles in turn are coupled with faster synchronous ripple oscillations (80–250 Hz) generated by hippocampal networks during non-declarative memory acquisition and during sleep (Chrobak and Buzsáki, 1998; Diekelmann and Born, 2010; Miyamoto et al., 2017) The increase in coupled activity between recurrently connected hippocampal and neocortical brain networks during sleep is thought to mediate the transfer or consolidation of memories. Accordingly, phase-coupling of slow wave, spindle, and ripple oscillations across cortical, thalamic, and hippocampal networks all are considered biomarkers of memory consolidation (Isomura et al., 2006; Staresina et al., 2015; Rothschild et al., 2017; Navarrete et al., 2020).

There is a growing interest in developing effective approaches to enhance slow oscillations and their coupling with spindles to improve memory consolidation in various clinical populations. Populations with altered spindle activity include schizophrenia (Wamsley et al., 2012; Stokes et al., 2022), Alzheimer's disease (De Gennaro and Ferrara, 2003; Rauchs et al., 2008; Weng et al., 2020), Autism Spectrum Disorders (Limoges et al., 2005; Tessier et al., 2015), sleep disorders (Leong et al., 2022) as well as natural aging (Martin et al., 2013; Mander et al., 2017; Purcell et al., 2017; Helfrich et al., 2018; Djonlagic et al., 2021). Historically, drugs have been developed to facilitate sleep physiology and memory. Multiple studies find the sleep aid, zolpidem, increases the density and power of sleep spindles (Dijk et al., 2010; Lundahl et al., 2012; Mednick et al., 2013; Niknazar et al., 2015; Zhang et al., 2020) and the coupling of slow wave and spindle oscillations (Niknazar et al., 2015; Zhang et al., 2020; Leong et al., 2022). Moreover, zolpidem strengthens hippocampal-prefrontal network coupling (Kersanté et al., 2023) and is correlated with improved memory task performance on the following day (Niknazar et al., 2015; Zhang et al., 2020; Leong et al., 2022). Unfortunately, zolpidem also enhances negative emotional memory consolidation and a meta-study finds zolpidem use is associated with increased suicide rates (Simon et al., 2021; Khan et al., 2022). Thus, zolpidem may only be an effective approach for enhancing slow waves, spindles, and memory in certain subpopulations. Multiple non-pharmacological approaches are under investigation including non-phase-locked or feedback-controlled transcranial electrical stimulation to increase slow wave oscillation amplitudes and improve overnight memory consolidation (Marshall et al., 2006; Lustenberger et al., 2016). Another approach is acoustic stimulation phase-locked to slow oscillations which enhances slow wave and spindle oscillations during sleep and can improve memory consolidation (Ngo et al., 2013b). Finally, a variation of the latter approach is to play sound as a contextual cue while people are learning and then play the same sound for “targeted memory reactivation” and consolidation during sleep (Cairney et al., 2016; Hu et al., 2020).

Over the past 10 years, fourteen studies have examined the efficacy of using acoustic stimulation during sleep to enhance slow wave and spindle oscillations and memory. One of the first studies to examine both the physiological and behavioral effects of overnight acoustic stimulation is a seminal study by Ngo et al. (2013b). This study plays sequences of two pink noise sound bursts phase-locked to the positive phase of an ongoing slow oscillation (SO) during the first 2 h of overnight NREM (stage 2, 3) sleep and measures how this impacts performance on a declarative word-pair memory task the next day (Ngo et al., 2013b). Here, the inter-stimulus interval between two noise bursts is set to each individual subject's average slow wave interval. Two outcome measures include the physiologic metric of SO amplitude and the behavioral metric of performance on a memory task. Positive outcomes include a significant increase in SO and spindle amplitudes and improved performance on a word-pair memory task with acoustic stimulation vs. control condition without stimulation (Ngo et al., 2013b). In this initial study, there is a substantial effect size (*d* = 1.08) supporting this non-pharmacological approach for improving verbal memory performance. In the past 10 years, seven clinical studies in total have examined the effects of phase-locked acoustic stimulation during sleep on word-pair memory in young adults or children (Ngo et al., 2013b, 2015; Ong et al., 2016; Leminen et al., 2017; Prehn-Kristensen et al., 2020). Five out of these seven studies find phase-locked acoustic stimulation during sleep increases SO amplitudes and improves performance on word-pair memory tasks in children (Prehn-Kristensen et al., 2020) and young adults that do not have diagnosed clinical conditions (Ngo et al., 2013b, 2015; Ong et al., 2016; Leminen et al., 2017). Two of the seven studies examining phase-locked acoustic stimulation effects on adult populations do not find significant improvements in word-pair memory (Henin et al., 2019; Schneider et al., 2020; Harrington et al., 2021). Similar to some prior studies (Henin et al., 2019; Diep et al., 2020; Prehn-Kristensen et al., 2020), the 2021 study by Harrington and colleagues uses a different type of word-pair memory test which requires subjects to remember unfamiliar word pairs. Based on this, they suggest that overnight SO phase-locked acoustic stimulation is more effective for consolidation (or reconsolidation) of familiar word pairs (Harrington et al., 2021). Similarly, other studies find phase-locked acoustic stimulation that significantly increases slow wave and spindle amplitudes does not improve performance on spatial memory (Henin et al., 2019), visual object and facial memory, or non-declarative finger tapping procedural memory tasks (Leminen et al., 2017). Given that different types of memory engage distinct brain areas and neuronal networks, it is possible that the current acoustic stimulation design is optimal for certain types of memory consolidation. To explore this possibility, Henin et al. (2019) set out to compare how phase-locked acoustic stimulation impacts both word-pair and spatial memory tasks using the same word-pair memory task employed by prior studies including that of Ngo and colleagues. Surprisingly, the Henin et al. (2019) study did not find a significant increase in performance with congruent or incongruent word-pairs in spite of observing significant increases in SO and spindle amplitudes for both. Collectively, there are five studies that support a phase-locked acoustic stimulation approach for enhancing declarative verbal memory but there remains considerable variability across all the studies completed to date.

Two recent meta-analytic studies (Wunderlin et al., 2021; Stanyer et al., 2022) examine potential study design moderators that could account for variability in effects across studies using the acoustic stimulation approach to enhance memory consolidations. Collectively, these meta-analytic studies consider age, phase-locking, and the type of memory task as potential contributors to variations in the efficacy of acoustic stimulation on memory. It is challenging to attribute the low effect size across studies to age alone because there are only three out of 14 current studies using older cohorts. For example, Papalambros et al. (2017) find a cohort of older adults (mean age 75.2 years) benefits from phase-locked acoustic stimulation showing improved verbal memory and substantial effect size (*d* = 0.63) on the order of that found with younger cohorts (Ngo et al., 2013b). In contrast, Schneider et al. (2020) find a cohort of older adults (mean age = 54.6 years) does not show enhanced memory performance following phase-locked acoustic stimulation. A *post-hoc* comparison by Schneider and colleagues finds that with or without acoustic stimulation SO amplitudes are smaller for older (mean age = 54.6 years) vs. younger (age 24.2 years) cohorts. Based on this result, Schneider and colleagues suggest the physiological capacity to generate and augment slow-wave oscillations in older populations may be reduced. The use of phase-locked vs. non-phase-locked acoustic stimulation is another potential contributor. However, there are not many studies probing memory effects with non-phase-locked stimulation or with older age cohorts. Indeed, Wunderlin and colleagues consider the combination of age and phase-locking as potential moderators for study outcomes. Accordingly, when combining studies using phase-locked acoustic stimulation and younger cohorts, the memory performance effect size increases almost two-fold larger than the overall mean (*d* = 0.25, 95% CI [−0.02, 0.53 ] to *d* = 0.44, 95% CI [0.09, 0.79]). This suggests that phase-locked acoustic stimulation in younger adults could be an effective tool to modulate memory retention. Toward this end, Wunderlin and colleagues compare portable systems that allow for multiple nights of electroencephalogram (EEG) recordings and phase-locked acoustic stimulation delivery with the goal of collecting quality data over multiple nights to strengthen future studies (Zeller et al., 2023). A meta-analysis by Stanyer and colleagues reports a memory performance effect size (*d* = 0.68, 95% CI [0.06, 1.30]) that is almost three-fold larger than reported for a similar set of studies in the meta-analysis by Wunderlin and colleagues. Nevertheless, Stanyer and colleagues also recommend future studies should include repeated measures and larger sample sizes to reduce between-study heterogeneity and help the future development of this novel approach. Collectively, these recent meta-analyses point toward potential ways to optimize future acoustic stimulation studies designed to improve memory and other cognitive operations.

In this review, we perform a meta-analysis of fourteen clinical studies completed between 2013 and 2023 that deliver acoustic stimulation during NREM sleep and measure the effects on slow wave amplitude and memory task performance. Specifically, we aimed to address the following four research questions:

R1. What is the average effect of acoustic stimulation during slow-wave sleep on sleep-dependent changes in word-pair retention?
R2. Does the effect size change with subsequent replications?
R3. If there are changes in the reported effects in subsequent replications, is this attributable to any codable study characteristics (e.g., mean age of sample, semantic congruence of word pairs, blinding procedures, change in SO power)?
R4. Are word-pair cued-recall tasks reliable enough to demonstrate changes in memory retention if they are present?

In order to address these questions, first we reproduce and extend prior meta-analyses by including three additional datasets from two new publications. We confirm the positive memory effects reported previously by Wunderlin et al. (2021) when including the same subset of studies using phase-locked acoustic stimulation and non-elderly adults. Upon including two new studies, one of which includes children and a second that includes an incongruent word-pair task, we find a significant downward trend in the effects of phase-locked stimulation on memory performance between 2013 and 2023. We run a meta-regression model examining eight potential moderators driving this downward trend including age, gender, phase-locked condition, properties of memory tasks (word count and word congruence), sleep condition (overnight vs. nap), double-blind procedures, and physiological increase in slow waves. None of these study-level characteristics account for this trend. In addition, difference scores are known to be highly unreliable in cognitive performance assessments, including the Stroop test (Hedge et al., 2018). Here, we examine the test-retest reliability of the difference scores (pre-sleep vs. post-sleep) used in all these studies to measure changes in memory retention. We find that these difference scores have low reliability and a strong potential to decrease statistical power. Accordingly, we hypothesize that unreliable memory task performance difference scores likely contribute a large degree of heterogeneity across studies testing acoustic stimulus effects on memory. Finally, we discuss how the memory task score, statistical approach, sample size, and other study design features may be improved to strengthen future research investigating the efficacy of acoustic stimulation to boost slow waves, spindle oscillations, and improve memory consolidation.

## 2. Methods and materials

### 2.1. Study selection

**Figure 1:**
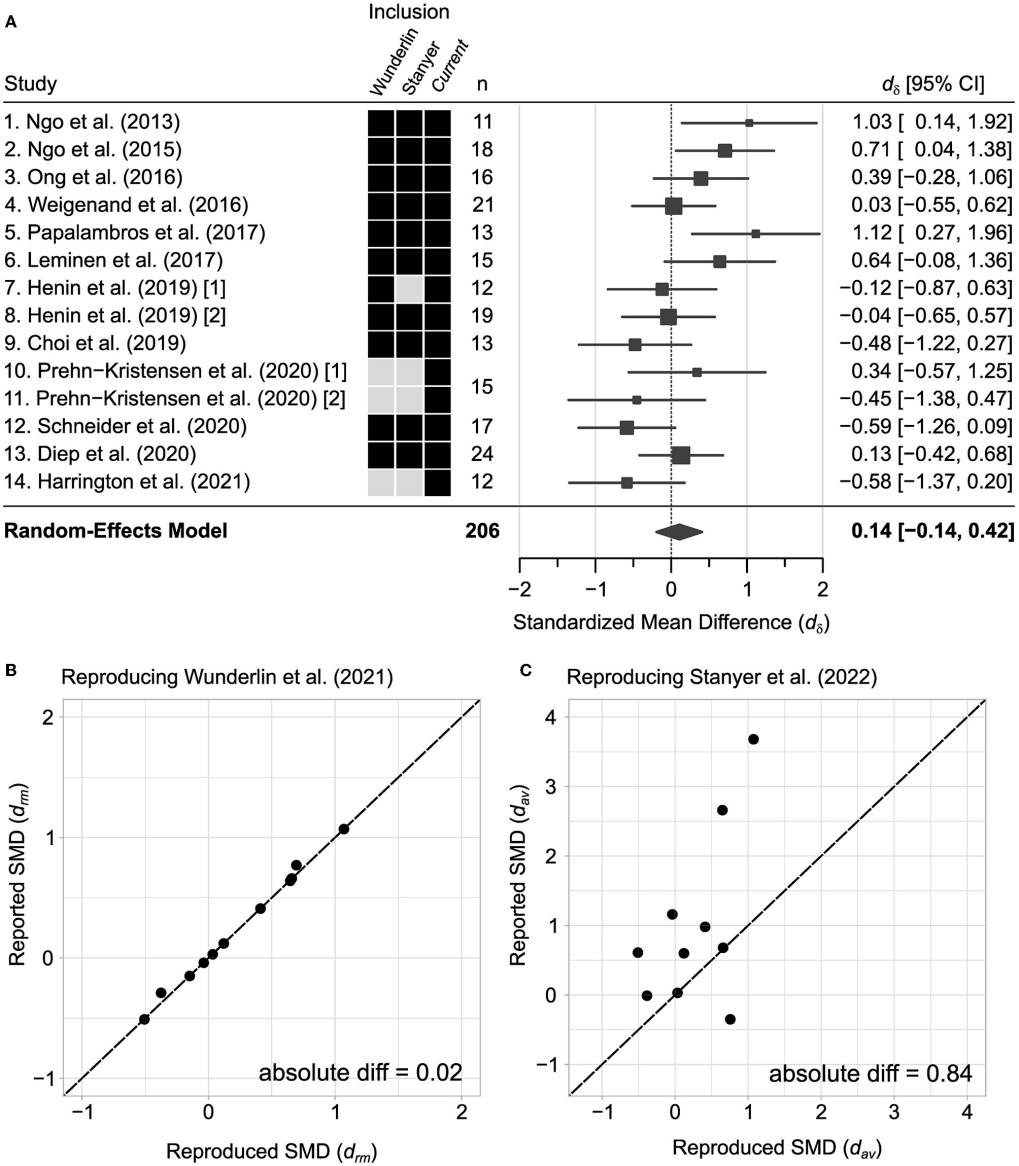
Standardized mean differences in the current meta-analysis and previous meta-analyses. **(A)** All effect sizes in the current meta-analysis estimated using Glass' estimator. **(B, C)** Standardized mean differences (SMD) reported from previous meta-analyses (Wunderlin et al., 2021; Stanyer et al., 2022) plotted against reproduced estimates using their respective methodologies. Purple lines indicate the mean of the reproduced and reported SMD where the intersection demonstrates how they diverge from equality.

The goal of the current meta-analysis is to confirm and extend the work of two previous meta-analytic reviews (Wunderlin et al., 2021; Stanyer et al., 2022). Here, we combine studies included in these two meta-analyses (Figure 1A). Additionally, we compare the statistical measures across our study and the two prior meta-analyses and confirm a high correspondence with Wunderlin and colleagues (Figure 1B) but less so with Stanyer and colleagues (Table 1 and Figure 1C). To expand on these prior studies, we conducted a supplemental google scholar search of the last 3 years (2020–2023), using the following key-word search: *(“acoustic stimulation” OR “auditory stimulation”) AND (“slow wave sleep” OR “slow oscillations”) AND (“memory” OR “consolidation”)*. The first 50 most relevant results are given a full-text screening for inclusion. This results in two relevant articles (Prehn-Kristensen et al., 2020; Harrington et al., 2021). Harrington et al. (2021) is likely excluded from previous meta-analyses (Wunderlin et al., 2021; Stanyer et al., 2022) as a consequence of being published after literature review. Prehn-Kristensen et al. (2020) may have been excluded as it compares subjects with and without attention deficit hyperactivity disorder (ADHD). Here, we extract the necessary effect size for the healthy control subjects for both the rewarded and non-rewarded conditions, which are included in the present meta-analysis. Additionally, we searched for studies that had cited the already-included articles and found no additional studies. In sum, we obtained *k*_*S*_ = 12 studies (*k*_*ES*_ = 14 effect sizes), and *N* = 206 healthy subjects of variable age (Supplementary Figure 1).

**Table 1:** Standardized mean difference estimates and reproduced estimates from previous meta-analyses.

	**Current (*d*_δ_)**	**Wunderlin (** ***d***_*****rm*****_ **)**			**Stanyer (** ***d***_*****av*****_ **)**		
**References**	**Estimate**	**Reported**	**Reproduced**	**Diff**	**Reported**	**Reproduced**	**Diff**
Ngo et al. (2013b)	1.03	1.07	1.07	0.00	3.68	1.07	2.61
Ngo et al. (2015)	0.71	0.64	0.65	0.01	2.66	0.65	2.01
Ong et al. (2016)	0.39	0.41	0.41	0.00	0.98	0.41	0.47
Weigenand et al. (2016)	0.03	0.03	0.03	0.00	0.03	0.03	0.00
Papalambros et al. (2017)	1.12	0.77	0.69	0.08	−0.35	0.76	1.11
Leminen et al. (2017)	0.64	0.66	0.66	0.00	0.68	0.66	0.02
Henin et al. (2019) [1]	−0.12	−0.15	−0.15	0.00		−0.15	
Henin et al. (2019) [2]	−0.04	−0.04	−0.04	0.00	1.16	−0.04	1.20
Choi et al. (2019)	−0.48	−0.29	−0.37	0.08	−0.01	−0.38	0.37
Prehn-Kristensen et al. (2020) [1]	0.34		0.23			0.24	
Prehn-Kristensen et al. (2020) [2]	−0.45		−0.41			−0.41	
Schneider et al. (2020)	−0.59	−0.51	−0.51	0.00	0.61	−0.51	0.10
Diep et al. (2020)	0.13	0.12	0.12	0.00	0.60	0.12	0.48
Harrington et al. (2021)	−0.58		−0.57			−0.57	
Mean	0.15	0.25	0.13	0.02	1.00	0.13	0.84

Effect size estimates in the current meta-analysis are compared against the reported effect sizes with previous meta-analyses. The reported effect sizes from Wunderlin et al. (2021) and Stanyer et al. (2022) are compared against our replication of their own calculations. The first column corresponds to our current use of Glass's estimator (*d*_δ_). The absolute differences between our reproduced estimates and the reported estimates from the original meta-analyses are listed under “Diff.” The mean of each column is an unweighted average of all elements.

### 2.2. Experimental study designs

Key study design features were aligned across all studies included. First, all studies included examined the efficacy of noise-burst acoustic stimulation during slow-wave sleep on overnight memory retention in the form of a word-pair cued recall task. Two experimental sessions consisted of a SHAM condition (control, no acoustic stimulation) and a STIM condition (with acoustic stimulation) during sleep (nap or overnight). The studies all had repeated measures and cross-over designs. That is, the same subjects participated in the control (SHAM, no audio) and acoustic stimulation (STIM) conditions. Ordering of STIM and SHAM conditions was appropriately counterbalanced in all studies. Word pairs were shown to participants, and they were subsequently assessed on their immediate recall prior to the sleep session. After waking, memory was assessed on the same word pairs. Memory retention scores were the difference in recall accuracy between pre-sleep and post-sleep assessments in all studies included. Memory retention scores were then compared between STIM and SHAM conditions.

### 2.3. Data collection

**Figure 2:**
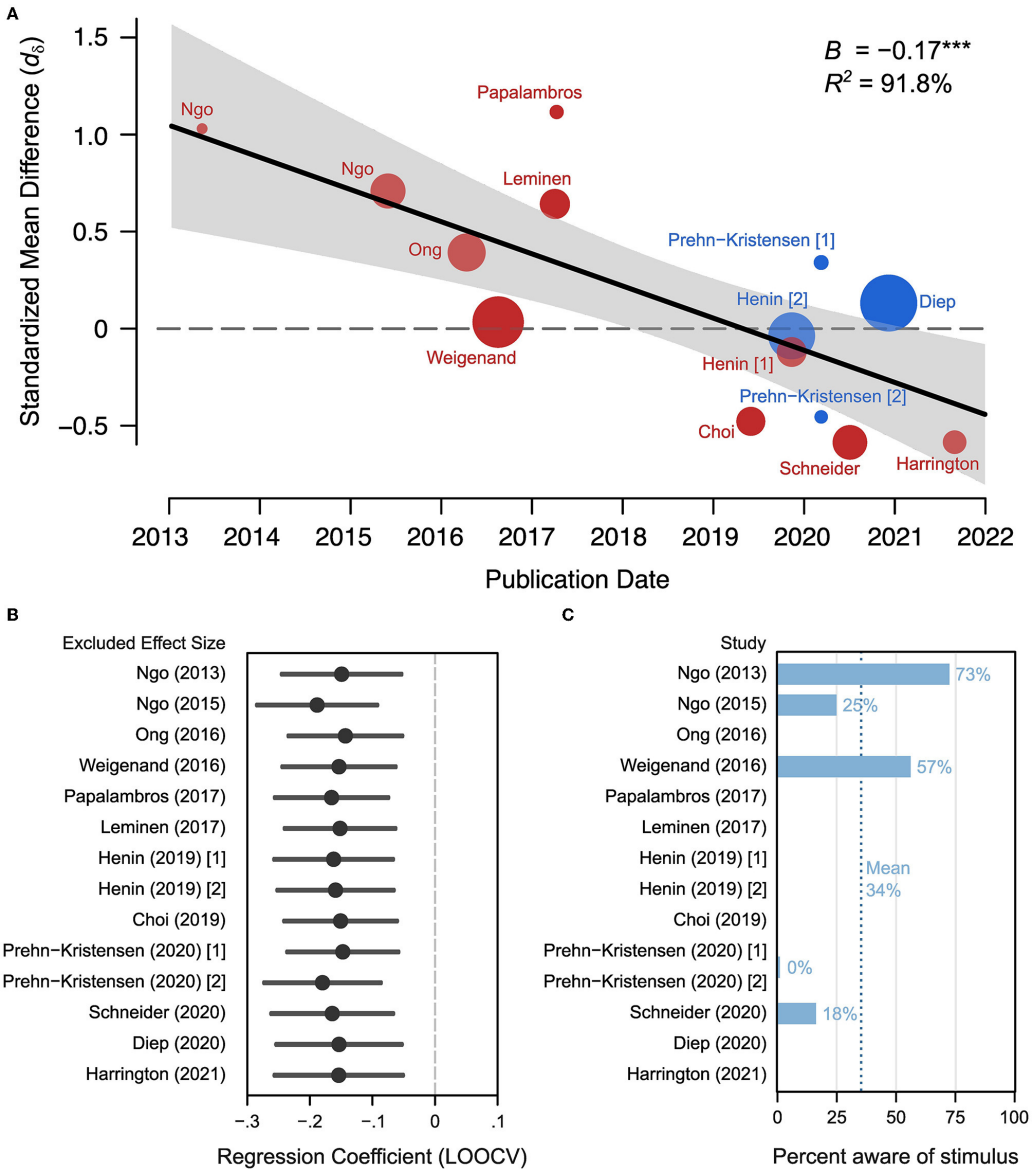
Effect decline and heterogeneity. **(A)** Meta-regression for publication date to observed effects across studies. Data-labels indicate first author of respective publication. **(B)** Results of a leave-one-out cross-validation (leave-one-out-cross-validation) where each regression coefficient is the fitted slope estimate when the corresponding study is removed. Note the resulting regression coefficients are similar indicating that no one study has major influence on the regression slope parameter. **(C)** The proportion of people reported hearing the auditory stimuli during the night when it was truly the STIM condition.

Data included in this meta-analysis was obtained from each publication or by contacting the corresponding authors. Relevant study characteristics were recorded (see Table 2) alongside effect size information. Fortunately, we were able to collect all characteristics of each moderator of each study. Most coded characteristics were directly reported in the respective manuscripts. However, slow oscillation power was not, therefore we calculated the standardized mean difference (SMD) in slow oscillation power/amplitude between STIM and SHAM. To quantify the effect of acoustic stimulation on memory retention, the standardized mean differences in word-pair retention scores between STIM and SHAM conditions were recorded. To obtain repeated measures effect sizes, correlation coefficients must be calculated between pre-sleep and post-sleep measurements. Since most studies did not report pre-sleep vs. post-sleep correlations, corresponding authors were contacted in order to acquire raw data, or data was extracted from figures containing sufficient information using WebPlotDigitizer. We were able to obtain raw data sets for seven independent samples from five studies (Ngo et al., 2013b, 2015; Weigenand et al., 2016; Henin et al., 2019; Schneider et al., 2020). Two samples were included from Ngo et al. (2015), but the second experiment had no SHAM condition therefore this sample is not used in the effect size calculations, however it is included in the reliability section of the meta-analysis. Since each study implemented a crossover design, we were able to estimate reliability coefficients for each of the seven raw data sets. Additionally, five studies reported the proportion of subjects that were aware of the stimulus at some point during the STIM condition (Ngo et al., 2013b, 2015; Weigenand et al., 2016; Prehn-Kristensen et al., 2020; Schneider et al., 2020). This proportion of subjects aware of the acoustic stimulus at any point was extracted from these five studies to examine the possibility that subjects were potentially not entirely blind to study conditions due to the no audio vs. acoustic stimulation designs employed by all (Figure 2C).

**Table 2:** Description of relevant moderators.

**Moderator**	**Description**
Publication date	Date of publication
Age	Mean age of participants
Proportion female	Proportion of participants that are female
Phase-locking	Phase-locked (1) or non-phase-locked (0) stimulation
Word count	Number of words during word-pair task
Congruent words	Semantically congruent (1) or incongruent (0) word-pairs
Overnight	Whole-night (1) or nap period (0)
Δ SO power	Change in slow oscillation amplitude/power
Double-blind	Experimenter or Investigator blinding to experimental condition

Provides a brief description of each study-level moderator used in the meta-regression and subgroup analyses. A full description of all moderators for each study can be found at: https://osf.io/8b49k/.

### 2.4. Effect-size calculation

Previous meta-analyses reported different formulations of standardized mean differences (SMD) in word-pair retention scores between STIM and SHAM conditions. Wunderlin et al. (2021) used a repeated measures estimator (*d*_*rm*_), whereas Stanyer et al. (2022) presumably used an average variance estimator (*d*_*av*_). Our meta-analysis finds that variances in STIM and SHAM conditions violate the assumption of equal variances. This is evidenced by the average (*n*-weighted) variance ratio of 1.38 ($S_{\text{stim}}^{2}/S_{\text{sham}}^{2}$). This indicates that the STIM condition has a 38% higher variance than the SHAM condition. Therefore, we use the Glass' estimator (*d*_δ_) to calculate standardized mean difference (SMD) because it does not assume equal variances. An in-depth description of various effect-size calculations can be seen in Lakens (2013). All the estimators, *d*_*rm*_, *d*_*av*_, and *d*_δ_ only differ in how they are standardized. Thus, they all take the form of:


(1) $$\begin{array}{l} d=\frac{M_{\text{stim}}-M_{\text{sham}}}{S^{*}}\cdot J\left(n\right) \end{array}$$

Where *J*(*n*) is the small sample correction factor, $J\left(n\right)=1-\frac{3}{4n-5}$, and *M*_sham_ and *M*_stim_ correspond to the mean of the SHAM and STIM condition, respectively. The differences between estimators are in their calculation of the standardizer, S^*^, such that:


(2) $$\begin{array}{l} \text{for }d_{rm}\text{: }S^{*}=\sqrt{\frac{S_{\text{stim}}^{2}+S_{\text{sham}}^{2}-2r_{d}S_{\text{stim}}S_{\text{sham}}}{2\left(1-r_{d}\right)}} \end{array}$$

(3) $$\begin{array}{l} \text{for }d_{av}\text{: }S^{*}=\sqrt{\frac{S_{\text{sham}}^{2}+S_{\text{stim}}^{2}}{2}} \end{array}$$

(4) $$\begin{array}{l} \text{for }d_{\delta }\text{: }S^{*}=S_{\text{sham}} \end{array}$$

Where *S*_stim_ and *S*_sham_ indicates the standard deviation for STIM and SHAM, respectively. The associated standard errors (*se*) for each SMD estimator are as follows:


(5) $$\begin{array}{l} se_{rm}=J\left(n\right)\cdot \sqrt{\left(\frac{1}{n}-\frac{d_{rm}^{2}}{2n}\right)\cdot 2\left(1-r_{d}\right)} \end{array}$$

(6) $$\begin{array}{l} se_{av}=J\left(n\right)\cdot \sqrt{\frac{2}{n}+\frac{d_{av}^{2}}{4n}} \end{array}$$

(7) $$\begin{array}{l} se_{\delta }=J\left(n\right)\cdot \sqrt{\frac{2}{n}+\frac{d_{\delta }^{2}}{2\left(n-1\right)}} \end{array}$$

Where *n* indicates the sample size. The point estimate and standard error of the repeated measures estimator (*d*_*rm*_) requires the correlation between STIM and SHAM retention scores (*r*_*d*_) which are rarely reported in repeated measure studies. Therefore, based on acquired raw data sets and plot digitizing (extracting data from figures), we calculated all available correlations. For the remaining studies where correlations were not available, the weighted mean correlation (i.e., random effects) is used to populate missing values.

### 2.5. Random-effects modeling

To perform our new meta-analysis and confirm prior meta-analyses and assess potential sources of variation in our regression model we estimate the true effect size using a Random-effects model approach (Figure 1A). Random-effects modeling is used to estimate the mean of true effect sizes. Random-effects models allow for variation between true effect sizes (heterogeneity) across studies as should be the case when studies vary in their population, methodology, or design. We use the restricted maximum likelihood estimator to calculate between-study heterogeneity, that is, the standard deviation of true effect sizes (τ). All meta-analytic modeling is completed in R using the *metafor* package (Viechtbauer, 2010).

### 2.6. Mixed-effects (meta-regression) modeling

A meta-regression model was implemented to quantify the potential moderating effects of multiple study-level characteristics on the effect size (*d*_δ_). Because our initial analysis identified a downward trend in effect size with publication date (Figure 2A), the regression model was logistically set up to examine whether independent moderators could account for this decline. Thus, we built nine different regression models to investigate whether the effect size was conditionally dependent on the study's publication date or other moderating variables (e.g., age; see Tables 2, 3). For interpretability, we also report the effects for various subgroups based off of categorical moderators (Table 4). The first model consists of a single moderator (publication date) while the other models consist of both publication date and one other moderating variable. As detailed above, the restricted maximum likelihood estimation method was used for all meta-analytic models. Additionally, we utilized the moving constant technique (Johnson and Huedo-Medina, 2011) by setting the initial moderator value to zero, this allows the intercept to be interpreted as the predicted effect size (*d*_δ_) at the date of the original publication (Ngo et al., 2013b). To compare the nine regression models, each model is re-fit with maximum likelihood (as opposed to restricted maximum likelihood) which is necessary to conduct a likelihood ratio test between models. The likelihood ratio test assesses whether the addition of a given model parameter is warranted.

**Table 3:** Meta-regression models and summary statistics.

**Model**	**1**	**2**	**3**	**4**	**5**	**6**	**7**	**8**	**9**
Intercept	0.989^***^ [0.492, 1.485]	0.758* [0.164, 1.352]	1.072** [0.358, 1.786]	0.875** [0.220, 1.531]	0.970* [0.020, 1.919]	1.415** [0.537, 2.294]	0.928** [0.244, 1.612]	0.915* [0.070, 1.560]	1.471^***^ [0.696, 2.247]
Publication date	-0.166^***^ [-0.254, -0.078]	-0.171^***^ [-0.257, -0.084]	-0.174^***^ [-0.273, -0.074]	-0.164^***^ [-0.257, -0.072]	-0.167^***^ [-0.261, -0.072]	-0.207^***^ [-0.318, -0.096]	-0.169^***^ [-0.261, -0.077]	-0.155^**^ [-0.252, -0.058]	-0.206^***^ [-0.305, -0.107]
**Additional moderators**									
+ Age		-0.393 [-0.880, 0.094]							
+ Proportion female			0.009 [-0.004, 0.022]						
+ Phase-locking				-0.113 [-0.844, 0.618]					
+ Word count					0.145 [-0.325, 0.615]				
+ Congruent words						0.0003 [-0.007, 0.008]			
+ Overnight							-0.318 [-0.854, 0.219]		
+ Δ SO power								0.091 [-0.500, 0.681]	
+ Double-blind									0.092 [-0.190, 0.374]
*R*^2^(%)	91.8	96.0	86.5	83.0	83.8	98.4	84.6	87.7	100
τ_resid_	0.110	0.077	0.142	0.159	0.155	0.049	0.151	0.135	0.000
*Q*_*m*_(*df*)	13.72 (1)	15.85 (2)	13.27 (2)	13.21 (2)	12.92 (2)	15.79 (2)	13.09 (2)	13.72 (2)	17.14 (2)
AIC	16.56	16.82	18.41	18.19	18.56	17.18	18.46	18.12	16.06
BIC	18.48	19.38	20.97	20.75	21.12	19.73	21.02	20.67	18.62
AICc	18.96	21.27	22.85	22.64	23.00	21.62	22.91	22.56	20.50
Likelihood ratio	–	1.740	0.152	0.369	0.001	1.385	0.100	0.444	2.502
p (LRT from model 1)	–	0.187	0.697	0.543	0.977	0.239	0.752	0.505	0.114

All models contain 14 effect sizes (*n* = 206 subjects). The regression coefficients with corresponding 95% confidence intervals are displayed alongside respective moderator variable. Each of the eight models contain publication date, with each successive model containing a single additional moderator one at a time. The final rows denote summary statistics of the meta regression models (*Q*, *Q*_*m*_-statistic for moderators; *R*^2^, percent of heterogeneity accounted for; τ_*resid*_, residual heterogeneity; AIC, Akaike information criterion; BIC, Bayesian information criterion; AICc, corrected Akaike information criterion). Bold AIC and BIC indicate lowest values (best model). Each column denotes the respective model's coefficients and summary statistics.

* *p* < 0.05,

** *p* < 0.01,

*** *p* < 0.001.

**Table 4:** Subgroup analyses.

				**95% CI**			
**Study**	*k*_*ES*_	*n*	${\bar{d}}_{\delta }$	**Lower**	**Upper**	τ	*I* ^2^
Overall	14	206	0.14	−0.14	0.42	0.385	52.8
**Age**							
Young	11	152	0.14	−0.16	0.43	0.319	42.4
Middle-Age/Elderly	3	54	0.19	−0.74	1.12	0.738	82.0
**Blinding**							
Single blind	10	148	0.20	−0.19	0.58	0.503	65.3
Double blind	4	58	0.03	−0.32	0.37	0.000	0
**Semantic congruence of word-pairs**							
Congruent	9	143	0.28	−0.11	0.67	0.466	63.3
Incongruent	5	63	−0.09	−0.42	0.24	0.000	0
**Sleep type**							
Whole night	12	178	0.14	−0.19	0.47	0.442	59.6
Nap	2	28	0.16	−0.34	0.66	0.007	0.04
**Phase locking**							
Phase locked	11	148	0.21	−0.14	0.57	0.461	59.2
Non-phase locked	3	58	−0.04	−0.39	0.31	0.000	0

*k*_*ES*_, number of effect sizes; *n*, total sample size; ${\bar{d}}_{\delta }$, mean standardized mean difference; τ, standard deviation of true effect sizes (i.e., heterogeneity); *I*^2^, Percentage of total variation in effect sizes due to variation in true effect sizes.

In addition, the sparsity of study-level characteristics could potentially drive our analyses to be insensitive to additive contributions of study-level characteristics to the observed heterogeneity in effect sizes. Many studies differed from Ngo et al. (2013b) methodologically, and some of those studies differed in multiple facets. To address this, we have constructed a “Methodological Deviation” factor consisting of the sum score of four other coded study design characteristics. Specifically, the sum score is taken as the sum of how study-level characteristics diverge from the seminal publication (Ngo et al., 2013b). The four characteristics used are age, semantic congruence, word count, and phase-locking. For example, if a study included subjects with similar mean age as Ngo et al. (2013b) (young adults), that component of general risk “Methodological Deviation” was coded as zero. The maximum “Methodological Deviation” is four, being relatively methodologically inconsistent with Ngo et al. (2013b), and the minimum zero being highly methodologically consistent with Ngo et al. (2013b). Subsequently, the “Methodological Deviation” factor was run with the omission of Ngo et al. (2013b). A major limitation to this index is that, since it is simply a sum score, it assumes that each methodological deviation from Ngo et al. (2013b) has identical weights.

### 2.7. Sensitivity analysis

Outliers can cause spurious results in small sample meta-regressions, therefore a leave-one-out cross-validation procedure was used to assess the robustness of the meta-regression model. For each iteration, one effect size is removed from the data set and the model is fit to the remaining effect sizes (Figure 2B). If the meta-regression model is to be considered robust, then each iteration of the leave-one-out-cross-validation should not greatly impact the model parameters. Specifically, each iteration of the leave-one-out-cross-validation should yield a significant slope coefficient, and no slope coefficient should significantly differ from the full model.

### 2.8. Meta-analysis of reliability coefficients

**Figure 3:**
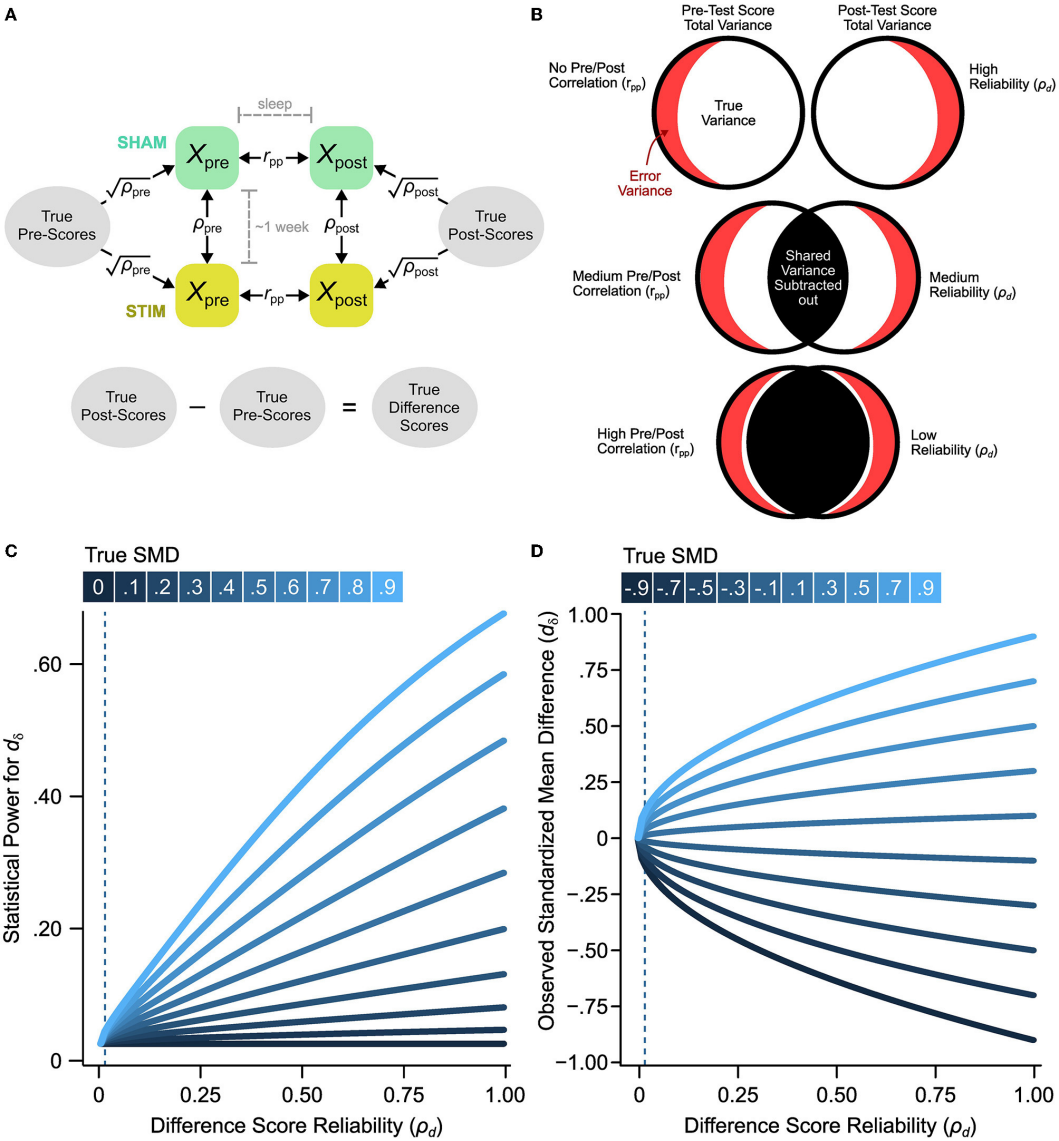
The effect of reliability on significance tests and observed effect sizes. **(A)** Measurement model of experimental paradigm indicate that true pre and post-sleep scores (unobserved scores uncontaminated by measurement error) underlies the observed pre-sleep and post-sleep scores. Differences in true scores between delayed and immediate recall are indicative of memory retention. **(B)** Venn diagram representation of how pre-post test correlation effects the reliability of difference scores. **(C)** For variable true effect sizes with a median sample size of ñ = 15, the statistical power increases with the reliability of the measure. The dashed lines indicate the estimated reliability of the word-pair recall difference scores. **(D)** The biasing effect of low reliability on observed SMDs (*d*_δ_) demonstrates that decreases in reliability bias SMDs toward zero. The effect of this bias is stronger in higher SMDs. The dashed lines indicate the estimated reliability of the word-pair recall difference scores.

Reliability is a psychometric property that indexes the precision of a measurement instrument. A test-retest reliability coefficient is a method of estimating the reliability of a task by administering the same task on two separate occasions and then calculated the correlation between time points (see a diagrammatic representation in Figures 3A, B). In all the experiments within this meta-analysis, subjects underwent a cross-over design, that is, the same participants were in the control (SHAM) and treatment (STIM) conditions. Since there are multiple measurements for each participant, this allows for the opportunity to assess the test-retest reliability of the word-pair recall task used to measure memory retention. The word-pair cued recall task is characterized by three phases: (1) A learning phase where participants are presented with a list of word pairs, (2) a pre-sleep (immediate) recall phase where participants are assessed on their word-pair recall accuracy and scored on the number of word-pairs they correctly recall, (3) a post-sleep recall phase where participants are assessed on their word-pair recall accuracy and scored on the number of word-pairs they correctly recall. Memory retention is scored by the number of word pairs correctly recalled post-sleep subtracted from the number of word pairs correctly recalled pre-sleep (see Figures 3A, B):


(8) $$\begin{array}{l} X_{d}=X_{\text{post}}-X_{\text{pre}} \end{array}$$

Where the difference score, *X*_*d*_ is used as the final memory retention score. However, word-pair recall is an imperfect measure of memory retention, therefore we can calculate the observed difference scores of *X*_*d*_ in terms of true scores (*T*; scores indicative of actual memory retention) and error variance (*E*; scores indicative of random error):


(9) $$\begin{array}{l} X_{d}=X_{\text{post}}-X_{\text{pre}}=\left(T_{\text{post}}+E_{\text{post}}\right)-\left(T_{\text{pre}}+E_{\text{pre}}\right) \end{array}$$

In classical test theory, the reliability of a measure is a ratio of true variance to observed variance such that:


(10) $$\begin{array}{l} \rho =\frac{S_{T}^{2}}{S_{X}^{2}}=\frac{S_{T}^{2}}{S_{T}^{2}+S_{E}^{2}} \end{array}$$

To compute the reliability of a difference score we need the reliability of pre-sleep scores (*ρ*_pre_), reliability of post-sleep scores (*ρ*_post_) and the correlation between pre and post-sleep scores (*r*_*pp*_). The reliability of pre-sleep (*ρ*_pre_) is the test-retest correlation between the SHAM condition's pre-sleep score and the STIM condition's pre-sleep scores. The reliability of post-sleep (*ρ*_post_) is the test-retest correlation between the SHAM condition's post-sleep score and the STIM condition's post-sleep scores. Lastly, the pre/post correlation (*r*_pp_) is estimated from the correlation between pre and post-sleep scores of the SHAM condition:


(11) $$\begin{array}{l} \rho_{d}=\frac{\rho_{\text{pre}}+\rho_{\text{post}}-2r_{\text{pp}}}{2\left(1-r_{\text{pp}}\right)} \end{array}$$

The estimates from each study are pooled to estimate the reliability of word-pair recall task as an indicator of memory retention (Table 5). A convenient property of Glass' estimator (*d*_δ_) is the simplicity of the correction for task reliability. For a discussion on corrections for repeated measures effect sizes see the blog post by Pustejovsky (2023). For *d*_δ_, the unreliability of the task introduces error variance into the total observed variance, thus inflating the observed standard deviation and subsequently reducing the observed standardized mean difference (*d*_δ, *obs*_). This attenuation can be formally defined as:


(12) $$\begin{array}{l} d_{\delta ,obs}=d_{\delta ,true}\cdot \sqrt{\rho_{d}} \end{array}$$

**Table 5:** Meta-analysis of test-retest reliability of word-pair cued recall task.

				**95% CI**			
**Coefficient**	*k*_*ES*_	*n*	**Est**	**Lower**	**Upper**	τ	*I* ^2^
Reliability of pre-sleep scores (*ρ*_pre_)	7	110	0.71	0.62	0.81	0.000	0
Reliability of post-sleep scores (*ρ*_post_)	7	110	0.84	0.77	0.90	0.000	0
Pre-post correlation (*r*_pp_)	7	110	0.95	0.92	0.98	0.030	64.1
Pre-post correlation—delayed ($r_{\text{p}}^{\left(del\right)}$)	7	110	0.74	0.65	0.83	0.000	0
Reliability of difference scores (*ρ*_*d*_)	7	110	0.01	0.00	0.20	0.000	0
Reliability of difference scores—liberal est ($\rho_{d}^{\left(lib\right)}$)	7	110	0.01	0.00	0.21	0.000	0
Observed correlation of difference scores (*r*_*d*_)	7	110	0.46	0.25	0.68	0.221	60.3

*k*_*ES*_, number of effect sizes; *n*, total sample size; Est, parameter estimate; τ, standard deviation of true effect sizes (i.e., heterogeneity); *I*^2^, Percentage of total variation in effect sizes due to variation in true effect sizes.

Where *d*_δ, *true*_ is the true standardized mean difference (see Figure 3C). This attenuation is also carried over to the standard error such that:


(13) $$\begin{array}{l} se_{\delta ,obs}=J\left(n\right)\cdot \sqrt{\frac{2}{n}+\frac{d_{\delta ,obs}^{2}}{2\left(n-1\right)}} \end{array}$$

The attenuation of the observed effect size (*d*_δ, *obs*_) and the corresponding change in the standard error (*se*_δ, *obs*_) altogether results in decreased statistical power with lower reliability (see Figure 3C).

## 3. Results

The aim of this meta-analysis is to compare and extend two previous meta-analyses (Wunderlin et al., 2021; Stanyer et al., 2022) with the goal of identifying ways to improve future studies on acoustic stimulation for memory enhancement. Our full analysis includes fourteen effect sizes from 12 studies that test how acoustic stimulation during sleep impacts post-sleep memory task performance (see Section 2, Figure 1). Our full analysis includes three additional effect sizes from two publications that followed the initial meta-analyses by Wunderlin and colleagues (Prehn-Kristensen et al., 2020; Harrington et al., 2021) (Figure 1A, boxes indicate distinct studies included for each meta-analysis). As a starting point, we first analyze a subset of 11 studies and confirm that our standardized mean difference effects are virtually identical to effect sizes reported by Wunderlin and colleagues (mean absolute difference in SMD estimates = 0.02; Table 1 and Figure 1B). Moreover, we confirm that the corresponding pooled effects for these eleven studies are small (*d*_*rm*_ = 0.25, 95% CI [−0.02, 0.53], *p* = 0.211), consistent with the prior report by Wunderlin and colleagues (*d*_*rm*_ = 0.25, 95% CI [−0.02, 0.53]). When analyzing a subset of six studies that employ phase-locked acoustic stimulation and include non-elderly adults, Wunderlin and colleagues report almost a two-fold increase in effect size (*d*_*rm*_ = 0.44, 95% CI [0.09, 0.79]). Accordingly, we confirm that phase-locked acoustic stimulation protocol and age have a significant positive result for this same subset of combined studies (*d*_*rm*_ = 0.44, 95% CI [0.07, 0.80]). However, this effect is no longer significant (*d*_δ_ = 0.23, 95% CI [−0.12, 0.58], *p* = 0.206) when we include three additional effect sizes for the current meta-analysis (Prehn-Kristensen et al., 2020; Harrington et al., 2021). As detailed below, our regression models find a progressive decline in effects over the past 10 years. This trend also explains away the subgroup effect previously reported by Wunderlin and colleagues for phase-locked stimulation in young adults (Wunderlin studies: *p* = 0.512; All current studies: *p* = 0.642), while the effect of publication date remains significant (Wunderlin studies: *p* = 0.002, All current studies: *p* < 0.0001). The Stanyer meta-analysis (Stanyer et al., 2022) examines the possible differences in effects for studies testing declarative vs. non-declarative memory. Stanyer and colleagues find no significant differences across memory task type and suggest that future studies should include more subjects and potentially more overnight measures which we concur. However, we find our standardized mean difference effects are not strongly correlated with those reported by Stanyer and colleagues (mean absolute difference in SMD estimates = 0.84; Figure 1C and Table 1). For our full meta-analysis, we examine the possibility that the inclusion of several recent studies that use double-blind procedures could account for the progressive decline in effect size (see Figure 2A, blue symbols). As observed for other moderators, double-blind procedures did not account for the decline in effect size (Table 3). Finally, we calculated the percentage of subjects that reported hearing acoustic stimuli in five independent studies (Section 2, Figure 2C). We estimate that 34% of participants report hearing acoustic stimuli at some point during their sleep session (*k*_*S*_ = 5 studies, 27 of 80 participants). This raises the possibility that many subjects in these studies are indeed not blinded to the experimental condition which could create placebo, or nocebo, effects (Petersen et al., 2014; Lindheimer et al., 2015; Faraone et al., 2022). This leads us to suggest that future studies should include a true placebo acoustic stimulation condition, as has been done for previous physiological studies (Ngo et al., 2013a).

### 3.1. Decline in observed effect sizes over time

Our meta-analysis examining a total of 14 studies finds a progressive annual decline in reported memory effects with acoustic stimulation during sleep. Accordingly, there is a decline in reported effects (standardized mean difference) between 2013 and 2023 (Figure 2A). Our meta-regression models examine multiple potential sources of variability contributing to this decline (Section 2) including publication date, age, gender, phase-locking, word count, and congruence of words in memory tasks, overnight sleep condition, double-blind procedures, and the physiological index of slow oscillation power (see Table 3). For effect sizes within categorical moderators, see Table 4. Using a meta-regression model (Section 2, Model 1) where publication date is the sole moderator, we find that the date of publication demonstrates a negative trend that accounts for 91.8% of the heterogeneity between studies (B = −0.166 [−0.254, −0.078], *Q*_*m*_ = 13.72, *p* = 0.0002, *k*_*ES*_ = 14 effect sizes, *n* = 206, τ_*resid*_ = 0.110, see Figure 2A and Table 3). Given the small number of effect sizes in this literature, we looked to evaluate the robustness of this trend with leave-one-out cross-validation (Section 2.7, Figure 2A) to ensure highly influential studies do not artifactually produce this trend. Our leave-one-out cross-validation (Section 2) analysis finds that the regression coefficient (beta) in each iteration remains significant and unchanged from the full model coefficient (Figure 2B). This indicates that the decline in effect size is not likely driven by outliers.

When adding one variable at a time using a step-wise procedure (Section 2), we find that no other study-level characteristic accounts for the annual decline in study effects (see Table 3, Models 2–9). Corrected Akaike and Bayesian Information Criterion (AICc and BIC, respectively) tests confirm that the publication date (Model 1) is the most parsimonious (according to BIC), explanatory model of the set (according to AICc). Additionally, log-likelihood test demonstrates that the addition of any alternative moderators does not improve the model fit. When we look at the predicted effect size (based on Model 1) across time, the first study (Ngo et al., 2013b) publication date has a large effect size *d*_δ_ = 0.99 (95% CI [0.49, 1.49]) that declines to a net negative effect of *d*_δ_ = −0.39 (95% CI [−0.73, −0.05]) in the year of the most recent publication (see Harrington et al., 2021, Figure 2A). This indicates that recent studies show a markedly reduced effect of acoustic stimulation on memory task performance, in spite of having similar cohort sizes. Next, we consider the possibility that there are cumulative methodological deviations accounting for the publication date effect. The relatively low number of studies deviating from Ngo et al. (2013b) in certain study characteristics (age, phase-locking, whole night) could lead to such analyses being underpowered for observing unique moderator effects. Likewise, combinations of study characteristics could drive heterogeneity in observed effect sizes. However, our “Methodological Deviation” moderator model (Section 2) indicates that cumulative methodological deviation does not account for any of the heterogeneity in effect sizes (B = −0.034 [−0.257, 0.190], *p* = 0.768). This indicates that a cumulative methodological deviation from Ngo et al. (2013b) does not explain the decline in effect sizes across studies or years.

### 3.2. Reliability of word-pair association retention task

In neuroscience, psychometric evaluations and measurement errors for assessing cognitive processes such as working memory and memory consolidation are rarely investigated. A potential shortcoming of standard test vs. retest memory task difference scores used in all the studies included in our meta-analysis is the loss of shared variability (Figure 3). For example, when there is no correlation between a first test (e.g., pre-sleep) vs. a second test (e.g., post-sleep) condition there is no shared variability resulting in a highly reliable difference score (Figure 3B, top row). In contrast, if subjects have similar variability across test and retest conditions there can be highly overlapped variability that is lost in the difference score measure (Figure 3B, rows 2 and 3). We estimate the reliability of the memory task difference scores as the ratio of true variance to total observed variance (Section 2, true plus error variance, Equation 10). If there is a loss in total observed variance due to overlapped variability for test vs. retest conditions this will result in low reliability for the memory performance difference score. The reliability of the word-pair association task used to measure memory retention (and consolidation) in this literature has yet to be evaluated. Thankfully, we can analyze this as investigators have provided seven raw data sets from a subset of five studies in the current meta-analysis (Section 2, Table 5). Each of these studies includes independent measures of memory task performance before and after sleep that can be used to calculate pre-sleep and post-sleep test-retest reliability (Section 2, Figure 3A). Using this model, we find that the pre-sleep memory task performance has fair reliability with a *ρ*_pre_ = 0.711 (95% CI [0.615, 0.807]). The post-sleep memory task performance shows a slightly better reliability of *ρ*_post_ = 0.835 (95% CI [0.775, 0.896]). However, the correlation between pre-sleep and post-sleep scores from the control sleep condition (SHAM acoustic stimulation) is notably strong at *r*_pp_ = 0.949 (95% CI [0.920, 0.979]). This high correlation between test-retest scores reduces the reliability of the difference score, as described above (Figure 3B). Additionally, we compute correlations for pre-sleep vs. post-sleep tests which are delayed by one week by calculating the correlation between pre-sleep scores from the STIM condition and post-sleep scores for the SHAM condition (Section 2). This delayed test-retest correlation is $r_{\text{pp}}^{\left(del\right)}$ = 0.742 (95% CI [0.655, 0.830]). Based on classical test theory (Section 2, Equation 7) our estimates indicate a near zero reliability for difference scores (*ρ*_d_ = 0.006, 95% CI [−0.188, 0.199]). Even if the delayed pre-sleep vs. post-sleep correlation is used (a more liberal estimate), the reliability of the difference score is still close to zero, $\rho_{d}^{\left(del\right)}$ = 0.015 (95% CI [−0.178, 0.208]). This indicates that the observed changes in word-pair retention may be attributable to noise. This is surprising considering that the correlation between difference scores for STIM vs. SHAM conditions is *r*_*d*_ = 0.465 (95% CI [0.247, 0.682]), as the difference score correlation should be bounded by the reliability estimate. However, even if we treat this correlation as an estimate of the reliability of difference scores, the reliability would still be considered very poor. In summary, measurement error in the task used to measure memory retention is expected to bias effect sizes and decrease statistical power (Figures 3C, D). It is possible that the poor reliability of the memory task difference scores in conjunction with low sample sizes and variable study-level characteristics could drive the observed heterogeneity in effect sizes.

## 4. Discussion

The use of acoustic simulation during slow-wave sleep to modulate slow waves and overnight word pair memory retention has been the focus of several studies, meta-analyses, and reviews within the last decade. Within this body of literature, there exists a variety of reported effects on memory performance, with meta-analyses suggesting small-to-moderate overall effect sizes and high degrees of variability across studies (Wunderlin et al., 2021; Stanyer et al., 2022). Previously, Wunderlin et al. (2021) found a larger memory enhancement effect when pooling studies that had included phase-locked acoustic stimulation and non-elderly participant age. For this same pool of studies, we confirm this result. Additionally, we confirm their report of a low overall effect and a large degree of heterogeneity even when controlling for acoustic stimulation and age. In addition, we extend the Wunderlin study by analyzing three additional recent studies and find there is no longer a significant pooled effect. Moreover, our meta-regression models examine multiple potential sources of variability including publication date, age, gender, phase-locking, word count and congruence of words in memory tasks, overnight sleep, double-blind procedures, and the physiological index of slow oscillation power (see Tables 2, 3). We find that none of these variables, except for publication date, accounts for the cumulative decline of memory effects with acoustic stimulation (see Table 3).

### 4.1. Publication date

Previous meta-analyses (Wunderlin et al., 2021; Stanyer et al., 2022) reported small-to-moderate effects of acoustic stimulation on sleep-dependent changes in word-pair memory retention as well as a large degree of heterogeneity unexplained. We demonstrate a yearly decline in reported effects, explaining 91.8% of the heterogeneity. In light of these findings, we also presented updated analyses of moderators examined in previous meta-analyses such as age and semantic congruence in word-pair memory tasks (Table 3). As expected, the addition of all other coded study characteristics did not explain heterogeneity above solely publication date. These trends describe a yearly decline in the reported efficacy of acoustic stimulation to modulate sleep-related word-pair memory consolidation (or retention). We also report that studies consistently demonstrate significant modulation of slow oscillation power ranging from *d*_δ_ = 0.48 to *d*_δ_ = 2.91. Heterogeneity in observed effects on memory performance was not attributable to that of observed effects of acoustic stimulation on slow oscillation power.

Decline effects are a common phenomenon in psychological sciences (Schooler, 2011; Pietschnig et al., 2019; Schimmack, 2020) and other disciplines (Munafò et al., 2007). The decline effect describes the phenomena where effect sizes decrease upon subsequent replications of the initial study. The yearly decline presented here likewise explains previously reported significant findings (Wunderlin et al., 2021) for acoustic stimulation overnight memory retention. The inclusion of publication date as a covariate in Model 6 by Wunderlin et al. (2021) diminished the effect of phase-locked stimulation in non-elderly where the regression coefficient is no longer significant. A finding which, is consistent still with the addition of recently published works with phase-locked acoustic stimulation in non-elderly (children and adults; Prehn-Kristensen et al., 2020; Harrington et al., 2021). This is further demonstrated to be robust to the contribution of outliers via our leave-one-out-cross-validation analysis (Figure 2B). Highlighting the consistency of the significant effect of publication date on the heterogeneity in observed effect sizes across studies.

There are many reported mechanisms for the Decline Effect. Recent work (Pietschnig et al., 2019) has demonstrated that underpowered designs of initial studies drive observed effect declines in intelligence literature. Contrary to this, as demonstrated through our leave-one-out-cross-validation analysis of the effect of publication date on the observed effect sizes across studies, we see that no single study drives this trend. A relevant factor here is that the presently analyzed studies do not vary greatly in their sample size (*n* range = 11–24) and therefore statistical power. In addition, other work has argued that unpublished findings contribute to effect declines across studies (Schooler, 2011), as well as for selective reporting (Schimmack, 2020). However, while we can not confirm any previously purported mechanisms driving effect declines here, we do suspect that the poor reliability of the task being used in the present literature does decrease statistical power and bias the effect size estimates (Figures 3C, D).

### 4.2. Task reliability

The measurement properties of behavioral and cognitive tests are rarely investigated in behavioral and cognitive neuroscience. In this literature, memory retention is measured using difference scores of word-pair recall tests (post-sleep scores minus pre-sleep scores). Difference scores are sometimes necessary to isolate cognitive processes such as inhibitory control which is often measured using a color-word congruence or Stroop test where the reaction times of congruent trials are subtracted from the reaction time of the incongruent word pairs to create a difference score. However, difference scores are notoriously unreliable (Rogosa and Willett, 1983; Hedge et al., 2018), especially when the pre-test and post-test is highly correlated, which is the case in this literature. Here, we find that the word-pair task expressed as a difference score is not a reliable index of memory retention. When calculating statistical power at different “true” effect sizes, we find that low difference score reliability is associated with lower power (Figure 3B, vertical dotted line). Additionally, this low difference score reliability can result in variable and biased observed effect size estimates as illustrated for a range of simulated true effect sizes (Figure 3D). With lower reliability, effect size estimates for *d*_δ_ and *d*_*av*_ are consistently biased toward zero. Taken together, it is evident that the reliability of overnight changes in word-pair retention is inadequate, and drives biased effect size estimates and decreases statistical power. Given we are only able to obtain reliability estimates for a small portion of the presently analyzed study, we can not with confidence evaluate study-level differences in reliability as a potential moderator. However, low task reliability could potentially account for the large heterogeneity in effects across studies.

### 4.3. Limitations

While the above analyses substantially explain a large degree of heterogeneity found in this body of literature, there remain other factors that could be of interest. There is great evidence suggesting that the rhythmic coordination of oscillations during slow-wave sleep is an essential component of memory processes during slow-wave sleep (Isomura et al., 2006; Staresina et al., 2015; Rothschild et al., 2017; Navarrete et al., 2020). It is our view that this is the theoretical and physiological basis for all the studies analyzed in the present study: Ngo et al. (2013b), Ngo et al. (2015), Ong et al. (2016), Leminen et al. (2017), Henin et al. (2019), Choi et al. (2019), Prehn-Kristensen et al. (2020), Schneider et al. (2020), Diep et al. (2020), and Harrington et al. (2021). Accordingly, the degree to which acoustic stimulation enhances slow-oscillation power and its temporal coupling to spindles could determine the corresponding memory consolidation. The frequency distribution of spindle oscillations and coupling of slow waves and spindle oscillations change during development through adulthood (Purcell et al., 2017; Hahn et al., 2020; Kurz et al., 2020). Additionally, slow wave activity declines with aging in adults (Mander et al., 2013, 2017). In theory, the efficacy of acoustic and other forms of stimulation to enhance slow waves, spindles, and memory consolidation could vary across development and aging. Hence, there is a strong rationale to constrain age to strengthen initial future studies validating an approach. Studies such as that of Schneider and colleagues find older adults can have reduced pre-stimulus slow wave oscillations compared with young adults and from a physiological standpoint this may impact the ability to enhance the slow waves (Schneider et al., 2020). However, the vast majority of studies fail to report the phase-locking estimates and their standard deviations for the arrival of acoustic stimuli relative to slow-oscillations, with the exception of three studies (Ong et al., 2016; Leminen et al., 2017; Papalambros et al., 2017). Similarly, sleep spindles are known to be coupled with slow oscillations and spindle density correlates with memory retention and general cognitive ability (Reynolds et al., 2018; Ujma, 2018) and spindles are considered biomarkers of the memory reconsolidation process (Hennies et al., 2016; Denis et al., 2021). Additionally, studies indicate slow and fast spindles play distinct roles in memory consolidation (Barakat et al., 2011; Mölle et al., 2011; Ayoub et al., 2013; Cox et al., 2017). In line with this, several studies included in this meta-analysis find distinct time scales of effects on slow vs. fast spindles (Ngo et al., 2013b; Ong et al., 2016; Weigenand et al., 2016; Henin et al., 2019) while other studies simply reported effects on fast spindles (Ngo et al., 2015; Leminen et al., 2017; Schneider et al., 2020; Harrington et al., 2021). However, several studies also reported reductions in sleep spindle power during periods of slow-wave sleep without acoustic stimulation (Weigenand et al., 2016; Diep et al., 2020; Schneider et al., 2020). Taken together, studies varied greatly in the extent to which they broadly characterized changes in these features during sleep. Inconsistent reporting of characteristic changes in slow wave and spindle oscillations and their relationships to one another and behavioral measures makes meta-analytically addressing these problems infeasible with such small sample sizes. In line with this, the sparse distribution of study-level characteristics in combination with these small sample sizes could lead to a lack of clear variability between study subgroups, and moderator analyses being insensitive to contributions of study-level characteristics to the heterogeneity. Thus, better tracking and reporting of these metrics in future studies with larger sample sizes would greatly facilitate future studies and meta-analyses.

### 4.4. Conclusions and recommendations for future research

In conclusion, our results demonstrate a yearly decline in the reported efficacy of acoustic stimulation to modulate overnight word-pair retention. The presence of such a trend in small bodies of studies investigating novel interventions is unsurprising (Schimmack, 2020). For the studies presented here, this finding is accompanied by another highly relevant characteristic across studies: the memory tasks used are largely unfit for the present study designs. While several study characteristics were unable to be accounted for in this meta-analysis, and the present literature analyzed is small, we find the results presented here suggestive of the following five recommendations:

- Current studies are underpowered due to small sample sizes. Future research should utilize larger samples to increase statistical power.
- Reliability of the memory task should be strengthened. Increasing the number of sleep and memory test sessions will provide repeated measurements that will enhance reliability, as reliability increases with the number of retests. Additionally, future studies could utilize latent change score modeling using data from three or more sessions, as this affords the ability to dissociate variance and to estimate the change in “true scores” between intervention and control conditions, thereby mitigating the statistical unreliability of memory task difference scores (Hedge et al., 2018).
- Provided steps are taken to ensure statistically robust measures and reliability, multiple forms of memory tasks may be suitable. For example, a meta-analysis finds acoustic, cue-related targeted memory reactivation enhances memory performance across 91 experiments that employ a variety of declarative and skill acquisition memory tasks. This approach likely taps into similar physiological circuits, given that targeted acoustic cues enhance memory when played during stages 2 and 3 of non-rapid-eye-movement (NREM) sleep but not during other stages of sleep (Hu et al., 2020).
- Our estimates suggest ~34% of subjects are not blind to the study conditions as they report hearing acoustic stimulation during the STIM condition. Many studies reported varying proportions of the participants becoming aware of acoustic stimuli during the evening. This raises the risk, as each study consisted of only a single STIM and SHAM session, that at least some subjects were cognoscente of when they were undergoing the experimental condition. The incorporation of a random-phase stimulation condition, similar to Ngo et al. (2013a), would be a simple yet effective way to provide a placebo-control in this regard.
- Mixed-reporting of any behavioral data or EEG features and characteristics hinders accurate accounting for variation in many characteristics (e.g., spindle features) across studies. All of which are research trajectories that could be greatly informative to a variety of disciplines. Future work should prioritize transparency by making their code and data (physiological and behavioral) publicly available (Wilkinson et al., 2016).

The application of these recommendations to future studies would greatly reduce the risk of small-sample bias, low statistical power, and confounding psychological effects. They would facilitate future research-synthesis efforts by providing the greater statistical power necessary for analyses of behavioral and physiological effects of acoustic stimulation of slow oscillations.

## Data availability statement

The original contributions presented in the study are publicly available. This data can be found here: https://osf.io/8b49k/.

## Author contributions

TH: conceptualization, data curation, methodology, formal analysis, writing—original draft, and writing—reviewing and editing. MJ: conceptualization, data curation, visualization, methodology, formal analysis, software, and writing—reviewing and editing. HR: funding, senior-authorship, and writing—reviewing and editing. JC: funding, senior-authorship, supervision, and writing—reviewing and editing. All authors contributed to the article and approved the submitted version.
