# Supplementary figures and images for: Memory retention following acoustic stimulation in slow-wave sleep: a meta-analytic review of replicability and measurement quality
Source: Front Sleep. 2023 May 11;2:1082253. doi: 10.3389/frsle.2023.1082253 (PMC12713896; doi:10.3389/frsle.2023.1082253)

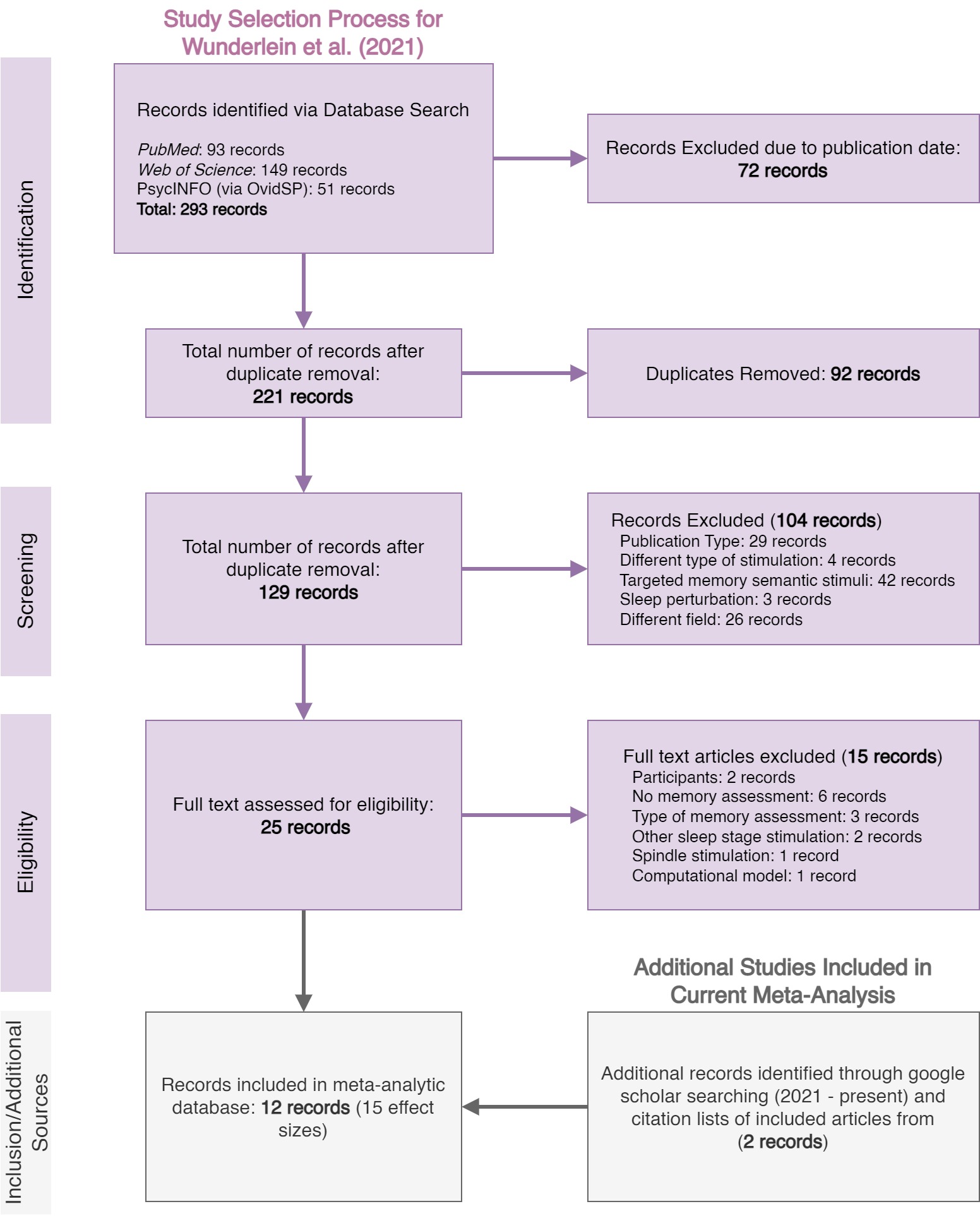

Supplement: Supplementary file 1 [file Image_1.JPEG]
